# PAIN IN PERSONS WITH DEMENTIA AND APATHY IN NURSING HOMES

**DOI:** 10.1093/geroni/igac059.1824

**Published:** 2022-12-20

**Authors:** Yo-Jen Liao, Ying-Ling Jao

**Affiliations:** Pennsylvania State University, Pennsylvania State University, Pennsylvania, United States; Pennsylvania State University, University Park, Pennsylvania, United States

## Abstract

Apathy and pain commonly occur in persons with dementia and significantly impact their quality of life. However, communication barriers in persons with dementia make pain assessment challenging. Apathy further complicates pain management in dementia due to decreased facial expression and verbal communication. This study aims to examine pain management in persons with dementia and apathy. This descriptive study included 13 residents with dementia and apathy from two nursing homes in Pennsylvania. Data on pain, pain-related diagnoses, and treatments were extracted from medical records. Participants’ mean age was 90 years old, and their mean apathy level was 54.6. All 13 participants had pain-related diagnoses with an average of 3.1 pain-related diagnoses (range=1-7). Four participants (30.7%) had pain reported in their medical records with osteoarthritis being the most common diagnosis (38.5%). Eight participants (61.5%) had pain-related diagnoses but did not have regular pain medication administered, and 3 of them (37.5%) had pain reported. In addition, five participants had one or more acute pain-related diagnoses, including surgery, fractures, and falls, and only 2 (40%) of them had pain reported. The average number of prescribed pain medications was 0.4 and 1.1 for regularly administered and as-needed medications, respectively. Acetaminophen was the most common administered medication. Overall, the results pointed out the potential issue that pain may be underrecognized and undermanaged in this population. More research is needed to examine the pain assessment and treatment in this population to promote pain management in persons with dementia and apathy.

